# The Role of Advanced Glycation End Products on Dyslipidemia

**DOI:** 10.3390/metabo13010077

**Published:** 2023-01-03

**Authors:** Jelena Vekic, Sanja Vujcic, Biljana Bufan, Dragana Bojanin, Khamis Al-Hashmi, Khaild Al-Rasadi, Anca Pantea Stoian, Aleksandra Zeljkovic, Manfredi Rizzo

**Affiliations:** 1 Department of Medical Biochemistry, University of Belgrade-Faculty of Pharmacy, 11000 Belgrade, Serbia; 2 Department of Microbiology and Immunology, University of Belgrade-Faculty of Pharmacy, 11000 Belgrade, Serbia; 3 Department for Clinical Chemistry and Hematology, Mother and Child Health Care Institute of Serbia “Dr Vukan Cupic”, 11000 Belgrade, Serbia; 4 College of Medicine and Health Sciences, Sultan Qaboos University, Muscat P.O. Box 373, Oman; 5 Department of Diabetes, Nutrition, and Metabolic Diseases, Carol Davila University of Medicine, 050474 Bucharest, Romania; 6 “Prof. Dr.N.C.Paulescu” National Institute of Diabetes, Nutrition and Metabolic Diseases, 050474 Bucharest, Romania; 7 Department of Health Promotion, Mother and Child Care, Internal Medicine and Medical Specialties, University of Palermo, 90100 Palermo, Italy

**Keywords:** AGEs, glycated LDL, atherogenic dyslipidemia, small, dense LDL, diabetes

## Abstract

Disorders of lipoprotein metabolism and glucose homeostasis are common consequences of insulin resistance and usually co-segregate in patients with metabolic syndrome and type 2 diabetes mellitus (DM). Insulin-resistant subjects are characterized by atherogenic dyslipidemia, a specific lipid pattern which includes hypertriglyceridemia, reduced high-density lipoprotein cholesterol level, and increased proportion of small, dense low-density lipoprotein (LDL). Chronic hyperglycemia favors the processes of non-enzymatic glycation, leading to the increased production of advanced glycation end products (AGEs). Apart from direct harmful effects, AGEs are also potent inducers of oxidative stress and inflammation. In addition, increased AGEs’ production may induce further qualitative modifications of small, dense LDL particles, converting them to glycated LDLs. These particles are even more atherogenic and may confer an increased cardiovascular risk. In this narrative review, we summarize the available evidence of the pathophysiological role and clinical importance of circulating AGEs and glycated LDLs in patients with dyslipidemia, particularly those with DM and related complications. In addition, we discuss recent advances and the issues that should be improved regarding laboratory assessment of AGEs and glycated LDLs, as well as the possibilities for their therapeutic modulation.

## 1. Introduction

Dyslipidemia is one of the most common metabolic disorders and a major modifiable risk factor for the development of atherosclerosis. In the vast majority of patients, alterations of serum lipid profile are associated with disorders of glucose metabolism, since both conditions share insulin resistance as the common underlying cause [1]. Insulin-resistant subjects are frequently characterized by the atherogenic lipid triad, involving increased triglycerides (TGs), reduced high-density lipoprotein cholesterol (HDL-C) levels, and elevated small, dense low-density lipoprotein (sdLDL) particles in plasma [2]. This lipoprotein phenotype is highly atherogenic and therefore is considered the most prominent risk factor for cardiovascular disease (CVD) development in patients with diabetes mellitus (DM) and metabolic syndrome [3]. 

Type 2 DM is a condition characterized by a hyperglycemia due to a relative insulin deficiency and peripheral insulin resistance [4]. Chronic hyperglycemia triggers multiple pathophysiological processes and affects the structure and function of virtually all biomolecules. One of the hallmarks of hyperglycemic conditions is non-enzymatic glycation, a process of glucose addition to proteins, lipids, or DNA that gives rise to the formation of advanced glycation end products (AGEs) [5]. In general, AGEs represent a heterogeneous group of metabolites with potent detrimental properties, which are considered to be contributors to the mechanisms behind the development of microvascular and macrovascular complications of DM. The accumulation of AGEs is also a common characteristic of ageing, while novel data suggest that AGEs play an active role in carcinogenesis [6]. 

The experimental data consistently showed that sdLDL particles are particularly vulnerable to adverse modification, with oxidation being one of the best studied processes [7]. Furthermore, evidence suggests that sdLDLs are more prone to non-enzymatic glycation, not only in patients with metabolic syndrome and DM [8], but also in subjects without DM [9]. While much of the literature focuses on the clinical importance of sdLDL and oxidized LDL particles, far less is known about glycated LDLs. Qualitatively modified LDL particles, either oxidized or glycated, have an active role in atherosclerotic plaque formation and progression [7]. In this respect, the determination of glycated LDLs could provide additional information on individuals’ CVD risk, beyond that provided by the LDL-C level. However, glycated LDLs are seldom evaluated in clinical practice. 

In this brief narrative review paper, we discuss the pathophysiological role and clinical importance of circulating AGEs and glycated LDLs’ determination in patients with dyslipidemia. Since several risk factors often cluster in patients with type 2 DM, we sought to address the association of AGEs with dyslipidemia, not only with standard lipid profile, but also with novel lipid biomarkers that changed the perspective of cardiometabolic risk, such is sdLDL. Taking into account the fact that AGEs represent important detrimental factors that may lead to adverse modifications of LDL particles, our idea was to consider whether the determination of AGEs as a causative factor or glycated LDLs as a consequence of non-enzymatic LDL glycation could be useful in preventing progression of cardiometabolic disorders. 

## 2. The Role of AGEs: Relationship with Dyslipidemia

### 2.1. Formation of AGEs

Biochemically, AGEs comprise a large group of different compounds produced by the non-enzymatic addition of reducing sugars and derivatives to amino groups of proteins, lipids, and nucleic acids [5]. Based on their physicochemical properties, AGEs are commonly classified into non-fluorescent AGEs, including carboxymethyl-lysine (CML), carboxyethyl-lysine (CEL), and pyrroline; and fluorescent AGEs, such as pentosidine and methylglyoxal-lysine dimer (MOLD) [10]. 

The accumulation of AGEs in human cells and blood is influenced by both exogenous intake and endogenous production. Endogenously, AGEs are synthesized in a series of reactions, known as the Maillard reaction [11]. In the first step of the reaction, unstable Schiff's bases are formed, which are further converted by Amadori rearrangement into stable ketoamines, i.e., AGEs’ precursors. These intermediaries can react with proteins or peptides, forming cross-links [11]. Subsequent reactions of oxidation, dehydration, and polymerization generate the final compounds known as AGEs [5,11]. Numerous factors, including age and DM, as well as exogenous sources, such as tobacco and dry heat-cooked food, may significantly increase AGEs’ production and, consequently, circulate AGEs’ pool [12,13,14,15]. 

### 2.2. Detrimental Effects of AGEs

**Figure 1:**
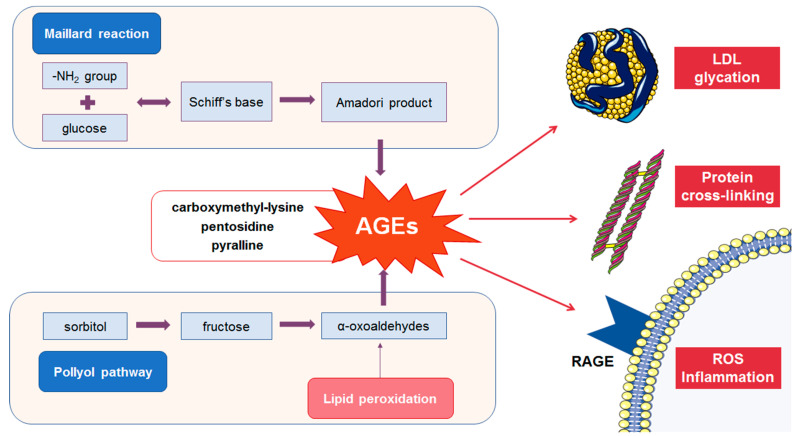
Main pathways of AGEs formation and major detrimental effects. The figure was composed by using Servier Medical Art templates, licensed under a Creative Common Attribution 3.0 (https://smart.servier.com, accessed on 14 December 2022.).

AGEs exert major detrimental effects by activating receptors for advanced glycation end products (RAGEs) or by cross-linking of the proteins (Figure 1), thus leading to the loss of their function [16,17]. The RAGE receptor belongs to the immunoglobulin superfamily of proteins and occurs in several isoforms with different roles. Cell-surface RAGE is a multi-ligand receptor that mediates signal transduction cascade involved in the inflammatory response [18]. Soluble RAGE isoforms (sRAGE) encompass a heterogeneous group of plasma proteins, formed by proteolytic cleavage of membrane-bound RAGE [19]. Furthermore, the circulating RAGE pool includes endogenous secretory RAGE (esRAGE), which is formed by alternative gene splicing and secreted from the cells [20]. These soluble isoforms are able to competitively bind the ligands, thereby interrupting their binding to the cell-surface RAGE receptors. In recent years, soluble RAGE isoforms, as well as the plasma AGEs/soluble RAGE ratio, are considered to be emerging risk factors for various diseases [21,22].

Cell-surface RAGE is presented in different cell types, including endothelial and immune cells [23]. Under physiological conditions, cellular expression of RAGE is low, but it can increase significantly in cardiometabolic disorders [24]. DM is characterized by the “metabolic memory”, i.e., excessive accumulation of AGEs, and this leads to overexpression of RAGE and persistent activation of the AGEs/RAGE axis [25]. This interaction triggers the NF-κB signaling pathway, leading to increased production of pro-inflammatory cytokines, adhesion molecules, prothrombotic and profibrotic factors, and reactive oxygen species (ROS) [26]. As a result, activation of the AGEs–RAGE axis stimulates angiogenesis, oxidative stress, cell proliferation, and apoptosis [16,17], which contribute to the development of microvascular and macrovascular complications. An increased expression of RAGE was also found in atherosclerotic plaque, suggesting their active role in the formation and progression of the lesion [27].

In recent years, the potential use of AGEs as a biomarker of DM and its complications has been studied, but the data are limited and inconclusive. Yozgatli et al. [28] reported an association between skin tissue AGEs, assessed by measuring skin autofluorescence, and the development of macrovascular, but not microvascular complications of DM. Other studies confirmed increased skin autofluorescence in DM patients, as well as its predictive value for neuropathy and nephropathy development [29,30]. Sternberg et al. [31] suggested that fluorescent AGEs in the skin could represent a good predictor of retinopathy progression. There is also evidence that AGEs affect bone metabolism in patients with DM [32]. Additionally, one study showed that skin autofluorescence better predicted cardiovascular mortality, as compared to glycated hemoglobin level [33]. Some authors reported an increase of specific serum AGEs, including CML in patients with both type 2 DM and CVD [34], while no correlation with other investigated parameters was observed. In contrast, total serum AGEs’ content did not differ between women with gestational DM and controls [35]. The observed inconsistency among the results could be a consequence of different methodological approaches for the quantification of AGEs, which require further improvements and harmonization. 

### 2.3. Assessment of AGEs in Biological Samples

Since AGEs represent a heterogeneous group of compounds, their quantification and comparison of the results obtained in different laboratories is complex. At present, several different methodological approaches are employed for the assessment of AGEs in biological samples (Table 1). However, there is still no reference method or universally accepted units of measurement.

Chromatography methods for AGEs quantification do not require the special pretreatment of samples and include HPLC with fluorescence detection, LC–MS/MS, and GC–MS [36,37]. These sophisticated techniques have high specificity and sensitivity but limited clinical use. Another approach is based on the ability of certain AGEs’ metabolites to emit fluorescence [38]. This method is simple and cost-effective and could be suggested for the rapid screening of serum AGEs content. It should be emphasized that only fluorescent AGEs can be measured, and other substances that are able to affect fluorescence (emit or extinguish it) might interfere during the determination. Immunochemical methods are often used for the detection of AGEs-modified proteins [39]. However, due to insufficient specificity of antibodies, the methods are subjected to interferences from protein adducts and other AGE species. Currently, several ELISA assays for the quantification of specific AGEs’ metabolites, such as CML [40], are commercially available and have a potential for clinical application. In addition to serum, the accumulation of AGEs in the skin can be assessed by a rapid, simple, and noninvasive technique that measures fluorescence emitted by AGEs cross-linked to collagen and other extracellular matrix proteins, following excitation by a low-intensity UV light [41]. The results obtained by the skin autofluorescence method correlate with AGEs’ levels in skin-biopsy specimens [42]. In that sense, skin autofluorescence measurement could be a valuable point-of-care technique for the assessment of tissue AGEs’ level, but more data are needed to verify the clinical importance of this biomarker in the prevention and management of DM complications. 

### 2.4. Relationship between AGEs and Dyslipidemia in Patients with DM 

Enhanced AGE formation and dyslipidemia usually co-segregate in patients with type 2 DM, particularly in those with inadequate metabolic control. However, associations of lipid status parameters with total plasma AGEs and/or specific AGE metabolites’ levels were only recently evaluated in DM patients. The study by Chang et al. [43] demonstrated that diabetic patients with increased AGEs in plasma were also characterized by having a worse lipid profile. On the other side, Rezaei et al. [44] recently showed an increase of total AGEs in plasma of patients with low HDL-C, but they found no significant changes in patients with elevated LDL-C and TG. Similarly, Indyk et al. [45] reported an inverse relationship between the level of HDL-C and concentration of melibiose-derived glycation product (MAGE), while there was no correlation between MAGE and the levels of total cholesterol and LDL-C. Both increased circulating AGEs [43] and higher AGEs accumulation in the skin [46] were positively associated with hypertriglyceridemia. The presented data suggest that advanced lipid testing is needed to assess the quality of lipoprotein particles and reveal the potential additive effects of AGEs and dyslipidemia on the risk for chronic complications of DM.

There is no doubt that elevated LDL-C concentrations are strongly associated with increased cardiovascular morbidity and mortality [47]. However, since LDL particles are highly heterogeneous in terms of their size and cholesterol content, a more detailed insight into their qualitative properties is frequently required. In certain individuals, the discordance between actual LDL-C level and a number of pro-atherogenic LDL particles was observed. Accordingly, a patient with optimal LDL-C but an increased level of small cholesterol-poor LDLs also carries a higher number of LDL particles, thus indicating a hidden cardiovascular risk [48]. An enhanced atherogenicity of sdLDL particles arises from delayed hepatic clearance, which increases their residence time in plasma and subsequent accumulation and oxidative modification in subendothelial space. Furthermore, newer research indicates that the altered proteome and lipidome of smaller LDL particles also influence their atherogenic properties [49,50]. The formation of sdLDLs is favored in the insulin-resistant state, and it is driven by increased synthesis and delayed catabolism of TG-rich lipoproteins. Therefore, elevated sdLDL particles in plasma is a common finding in obese, metabolic syndrome, and type 2 DM patients, but it is also presented in patients with chronic kidney disease, chronic inflammatory, and endocrine disorders [7]. At present, the clinical importance of sdLDLs’ assessment is mainly reflected by their ability to reveal a residual CVD risk [3]. 

It should not be neglected that patients with dyslipidemia usually have suboptimal HDL-C levels, which is particularly evident in those bearing atherogenic lipoprotein phenotype [2]. Although HDL possesses potent cardioprotective effects—not only by means of cholesterol efflux and reverse cholesterol transport processes, but also through its antioxidant, anti-inflammatory, antithrombotic, antiapoptotic, and vasodilatory actions—a low HDL-C level is currently considered a biomarker of CVD risk, and not a therapeutic target [51]. The abovementioned protective functions are highly compromised in cardiometabolic diseases, as a consequence of qualitative alterations of HDL particles. Specifically, these patients have dysfunctional HDLs, which are smaller, denser, and cholesterol-poor, but enriched in TG and acute phase proteins [52]. In patients with DM, there is also a possibility for non-enzymatic glycation of HDL-associated proteins, such as apolipoprotein A-I and antioxidative enzyme paraoxonase 1 (PON1), and this process further affects their plasma level, activity, and ultimately overall HDL functionality [53]. Of note, adverse modifications of LDL particles are even more accelerated in these circumstances.

## 3. The Role of Glycated LDL: Relationships with Inflammation 

### 3.1. Formation and Detrimental Effects of Glycated LDL Particles

Adverse modifications of plasma lipoproteins are particularly relevant in patients with dyslipidemia, due to increased availability of substrates. In addition, the bulk of glucose and pro-oxidants in plasma of patients with DM accelerates lipoprotein glycation and oxidation. Since apolipoprotein B-100 (apoB) represents the single protein moiety within LDL, it undergoes the most significant changes upon glycation. This process subsequently affects the metabolism of LDL particles. Namely, the clearance of LDLs via hepatic LDL receptors is accomplished by the specific recognition of the lysine residues within the N-terminal end of apoB. The same lysine residues are the main target of glycation process, which consequently diminishes receptor-mediated uptake of LDL [54,55]. In addition to lysine, other amino-acid residues of apoB, such as arginine, are also prone to glycation [56]. As explained earlier, patients with DM are characterized by elevated sdLDL particles in plasma, as a consequence of their fostered formation and delayed clearance by the LDL receptors. Furthermore, it has been shown that the prevalence of sdLDLs is increasing alongside worsening of metabolic control [57]. Such an increased proportion of sdLDLs is frequently categorized as the LDL B phenotype, denoting elevated risk for CVD [58]. The results of the study by Sánchez-Quesada et al. [59] clearly showed that DM patients with LDL B phenotype had increased levels of glycated LDLs. 

**Figure 2:**
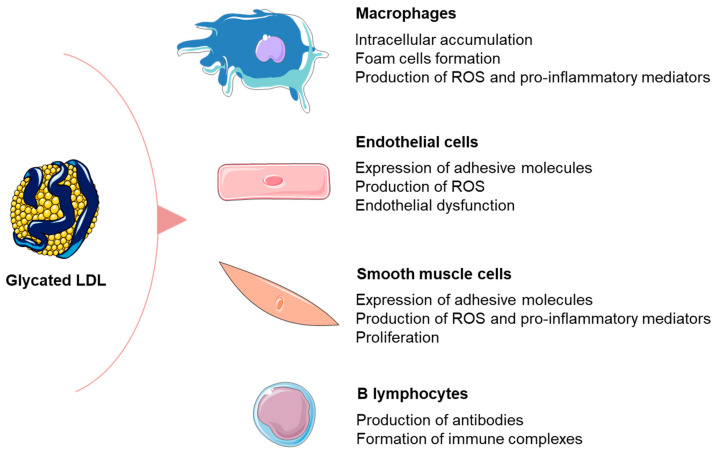
The effects of glycated LDL particles on vascular cells. The figure was composed by using Servier Medical Art templates, licensed under a Creative Common Attribution 3.0 (https://smart.servier.com, accessed on 14 December 2022.).

The accumulation of AGEs favors a cross-linking of extracellular matrix proteins [11]. In addition, data from in vitro studies demonstrated that the glycation of LDL particles enhances their binding to extracellular matrix proteoglycans, as well as their mutual aggregation [60]. Collectively, these processes form the basis for the prolonged retention of glycated LDLs in subendothelial space. In this scenario, glycated LDLs are increasingly recognized by scavenger receptors, preferentially by SR-A and CD36, and their subsequent accumulation in macrophages is able to elicit foam cell formation even without oxidative modification [61]. However, it should also be noted that glycated LDLs are potent inductors of ROS generation in different types of vascular cells [62,63], thereby increasing the potential for oxidative modifications. The study by Sima et al. [63] showed that glycated LDL particles have a significantly higher content of advanced glycation and lipid peroxidation end products, pentosidine and malondialdehyde, respectively, as compared to unmodified LDLs. Another important aspect that significantly contributes to atherogenicity of glycated LDL particles is the ability to promote synthesis of adhesive molecules and pro-inflammatory mediators in vascular cells [62,63]. Taken together, these observations provide a mechanistic explanation for the clinical importance of glycated LDLs in patients with dyslipidemia (Figure 2).

### 3.2. Relationship between Glycated LDL and Inflammation in Patients with DM

In general, the processes of LDL modification, including glycation, create different neoepitopes which are able to trigger autoimmune response [64,65,66]. As a consequence, modified LDL particles in plasma are mainly associated to specific autoantibodies, forming immune complexes [65]. However, the spectrum of the antibodies against glycated LDL is wide due to the heterogeneity of epitopes formed by the interaction of different AGEs metabolites with LDL, which limits the generalization of the results. Autoantibodies against modified LDL were detected in serum from both diabetic and non-diabetic subjects [64,65,67]. In particular, in patients with DM, increased serum levels of antibodies against methylglyoxal-modified LDL [64] and D-ribose-modified LDL [67] were reported. Khan et al. [64] recently showed that the level of IgG antibodies against methylglyoxal-modified LDL increases with DM duration, suggesting their potential involvement in the development of complications. On the other side, Mironova et al. [68] did not find a significant difference in the levels of anti-glycated LDL antibodies in serum of type 2 DM patients, coronary artery disease patients, and healthy controls. However, they found a significantly higher content of apoB and cholesterol within immune complexes isolated from diabetic patients that positively correlated with their ability to induce accumulation of cholesterol esters in macrophages [68].

Virella et al. [69] showed that the main isotype of antibodies against AGE–LDL in serum of type 1 DM patients is IgG, predominantly of IgG1 and IgG3 subclasses. The same group demonstrated that AGE–LDL autoantibodies recognize CML and CEL epitopes [69]. In vitro studies provided evidence that immune complexes containing modified LDL have several-fold higher pro-inflammatory potential than modified LDL [65]. It is well-known that IgG1 and IgG3 subclasses are potent triggers of immune system effector mechanisms such as the activation of the classical pathway of complement. They are also able to efficiently interact with most FcγR on the FcγR-expressing cells, resulting in phagocytosis [69,70]. In this respect, it is considered that immune complexes containing modified LDL may modulate inflammation in atherosclerosis by FcR signaling and complement activation [71]. In particular, it was suggested that, following the uptake by FcγR, immune complexes containing modified LDL contribute to the accumulation of cholesterol esters and subsequent activation of macrophages [72]. The process is facilitated by the products of complement system activation [71]. The mechanism involving FcγR was also proposed to mediate production of monocyte colony-stimulating factor [73] and components of connective tissue [74], which might contribute to cellular proliferation and fibrosis.

So far, several approaches have been employed to evaluate the extent of LDL glycation in plasma of patients with DM, but the most frequently reported forms were glycated apoB and AGE-modified LDLs. Irrespective of the used approach, the data consistently showed that patients with DM have significantly higher levels of glycated LDL particles than healthy subjects [59,75,76]. Furthermore, increased glycated apoB was associated with an approximately two-times-higher risk of myocardial infarction in elderly diabetic and non-diabetic subjects [77]. The results of Cohen et al. [75] showed a gradual increase of glycated LDL particles’ concentration in parallel with rise of albumin excretion rate, while Siddiqui and colleagues [78] found elevated glycated LDLs in patients with diabetic nephropathy. These findings suggest that glycated LDL particles might also be implicated in the development of microvascular complications of DM, although the available data are limited.

## 4. Implications for Cardiovascular Prevention and Future Directions

The clinical importance of glycated LDL particles should also be discussed from the perspective of risk management by therapeutic modulation. At present, statins are considered the first-line therapy for dyslipidemia. In line with the previous, Younis and colleagues [8] showed that the levels of glycated apoB in type 2 DM patients receiving statins were significantly lower than the levels in statin-naïve patients. Statin therapy has not only lipid-lowering action, but also exerts numerous pleiotropic effects. In this respect, the experimental data showed that statins may prevent the oxidation of glycated LDL particles and reduce levels of atherogenic small, dense LDL [79], while the results from the Protection Against Nephropathy in Diabetes with Atorvastatin (PANDA) trial demonstrated that atorvastatin treatment significantly reduced the levels of both glycated apoB and oxidized LDL in patients with type 2 DM [55]. Obviously, the process of LDL glycation in patients with DM can be delayed by achieving optimal metabolic control. The available evidence suggests that metformin, the most widely prescribed anti-diabetic medication, is able to decrease the level of reactive dicarbonyl compounds and prevent glycation of plasma lipoproteins [80]. Rabbani et al. [56] demonstrated a lesser extent of apoB glycation in type 2 DM patients receiving metformin as compared to patients who were not on metformin treatment. Furthermore, the addition of metformin to intensive insulin therapy reduced the levels of glycated LDLs in obese patients with type 1 DM [81]. These findings imply that improvements in both glycaemia and dyslipidemia act synergistically in reducing cardiovascular risk associated with glycated LDL in patients with DM. 

Dietary interventions and physical activity are the main strategies for cardiometabolic risk reduction. Numerous naturally occurring plant products have been suggested to prevent non-enzymatic glycation [15,82]. Moreover, recent studies reported a positive impact of adherence to the Mediterranean diet on serum AGEs levels [83], as well as on AGEs accumulated in the skin [84]. In line with the previous studies, the results provided by Lotan et al. [85] showed that reduced AGE intake may significantly decrease circulating AGEs levels in older patients with DM. Furthermore, a mounting body of evidence suggests that physical activity also has beneficial effects on both serum and AGE content in the skin [86,87,88], particularly in patients with cardiometabolic diseases [89]. On the other side, bariatric-surgery-induced weight loss was not associated with the significant regression of skin AGEs’ accumulation [90]. Bearing in mind the findings that decreased dietary AGEs are associated with lower levels of glycated LDLs in the plasma of patients with DM [91], as well as that energy-restricted weight loss is associated with reduced plasma AGEs levels [92], the importance of nutritional and lifestyle changes should not be neglected. More recently, a beneficial effect of bariatric surgery on the level of glycated apoB was reported during the follow-up of obese patients with and without type 2 DM [93].

**Figure 3:**
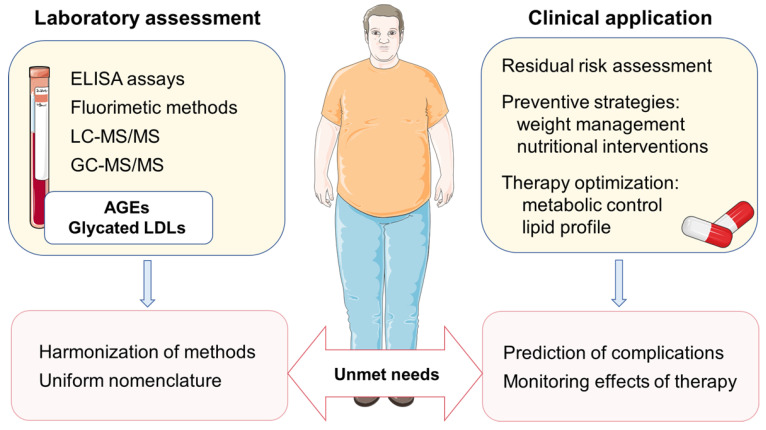
Potential applications of AGEs and glycated LDL particles in the prevention and management of cardiometabolic diseases. The figure was composed by using Servier Medical Art templates, licensed under a Creative Common Attribution 3.0 (https://smart.servier.com, accessed on 14 December 2022.).

At this point, it should be noted that AGEs are also involved in other ageing-associated diseases, and therefore the literature on clinical and epidemiological evidence regarding AGEs’ determination is steadily growing. More recent prospective data from Cardiovascular Health Study and Multi-Ethnic Study of Atherosclerosis cohorts showed that circulating AGEs were independent predictors of incident CVD in older adults, but not among younger subjects [94]. These findings indicate that the contribution of AGEs to CVD risk might be modulated by patients’ age and associated comorbidities, which should be confirmed in future studies. Another emerging topic of modern scientific investigations is whether novel markers of dyslipidemia, particularly modified LDLs, could improve prevention of cardiometabolic disorders. However, unlike sdLDL and oxidized LDL, the clinical importance of glycated LDLs’ determination is less certain. One potential explanation is the fact that, so far, a wide variety of commercially available or in-house developed assays were employed to assess the extent of LDL glycation across the studies [75,78,95,96]. Since these tests examined different properties of glycated LDLs, the terminology used across the studies is heterogeneous, thus limiting the generalization of the results and requiring harmonization (Figure 3). Another potential reason could be that other forms of modified LDLs were studied in more detail since these particles are also presented in other pathophysiological conditions [7]. Nevertheless, none of the modified LDL forms is interchangeable or reflected by the serum LDL-C level, which is particularly evident in insulin-resistant subjects [1] and patients with DM with optimal or low LDL-C levels [3,97]. Thus, a finding of increased glycated LDLs could guide future management of patients with innovative anti-diabetic therapies, improving both metabolic control and lipid profile [98,99], especially sdLDL levels [100]. 

Although the role of AGEs is still not fully elucidated and novel aspects of their metabolism and potential effects on receptors are still being revealed, a finding of high concentrations of AGEs in the serum or skin tissue of patients with DM indicates the need for more rigorous metabolic control, particularly in those with atherogenic dyslipidemia. The results of experimental and clinical studies demonstrated strong associations between increased AGE formation and atherosclerotic risk, but more data are needed to resolve all raised methodological issues and recommend their assessment in the routine clinical practice. As emphasized above, the process of non-enzymatic glycation of LDL particles further affects their atherogenic properties, with sdLDLs being more susceptible to such adverse modifications. Therefore, the assessment of glycated LDLs’ levels could provide additional clinical information on patients' risk beyond that estimated merely by the measurement of LDL-C concentration. In order to achieve this, one of the main tasks in the future should be the development of specific assays for the measurement of both circulatory AGEs and glycated LDL particles.

## 5. Conclusions

Despite the available evidence that non-enzymatic glycation and dyslipidemia might have a synergistic effect on the development of diabetic complications, future research is required to explore AGEs and glycated LDLs as biomarkers of DM progression, as such research may lead to novel diagnostics and therapeutic approaches. Nevertheless, the recent data on the beneficial effects of body weight reduction on plasma AGEs and glycated LDL particles are promising. These novel findings indicate that firmly established atheroprotective measures, such as healthy dietary patterns and exercise, might interfere with AGEs’ formation and consequent LDL glycation, thus offering new possibilities for preventive actions. In addition to the efforts to improve cardiometabolic health by positive changes of lifestyle habits, recent insights from clinical studies can also assist in the optimization of the therapy.

## Figures and Tables

**Table 1:** Methods for the assessment of AGEs in biological samples.

Method Principle	Benefits	Disadvantages
Fluorescence	Rapid Simple Non-expensive	Non-fluorescent AGEs are not measured Low sensitivity and specificityInterferences
Chromatography (HPLC-fluorescence; LC–MS/MS and GC–MS)	High sensitivity and specificity High accuracy Measurement of specific AGEs (CML, CEL, etc.)	Time-consuming High costs Qualified personnel required
Immunochemistry (ELISA, Western blot)	Simple Rapid Non-expensive	Only for measurement of protein–AGEs Low specificity and accuracy
